# Phlebotomy Treatment for Elimination of Perfluoroalkyl Acids in a Highly Exposed Family: A Retrospective Case-Series

**DOI:** 10.1371/journal.pone.0114295

**Published:** 2014-12-12

**Authors:** Stephen J. Genuis, Yanna Liu, Quentin I. T. Genuis, Jonathan W. Martin

**Affiliations:** 1 Faculty of Medicine and Dentistry. University of Alberta, Edmonton, Alberta, Canada; 2 Division of Analytical & Environmental Toxicology, Department of Laboratory Medicine & Pathology, University of Alberta, Edmonton, Alberta, Canada; 3 MD Program, Faculty of Medicine and Dentistry. University of Alberta, Edmonton, Alberta, Canada; Chiba University, Graduate School of Medicine, Japan

## Abstract

**Background:**

Perfluoroalkyl acids (PFAAs) are a family of commonly used synthetic chemicals that have become widespread environmental contaminants. In human serum, perfluorohexane sulfonate (PFHxS), perflurooctane sulfonate (PFOS), and perfluorooctanoate (PFOA) are most frequently detected, in part owing to their long elimination half-lives of between 3.8 yrs (PFOA) and 8.5 yrs (PFHxS). These PFAAs also cross the placenta and have been associated with developmental toxicity, and some are considered likely human carcinogens. Interventions to eliminate PFAAs in highly contaminated individuals would reduce future health risks, but minimal research has been conducted on methods to facilitate accelerated human clearance of these persistent substances.

**Methods:**

Six patients with elevated serum concentrations from a single family were treated by intermittent phlebotomy over a 4–5 year period at intervals similar to, or less frequent than what is done for routine blood donation at Canadian Blood Services. The apparent elimination half-life (HL_app_) for PFHxS, PFOS, and PFOA in this treated population was calculated in each patient and compared to the intrinsic elimination half-lives (HL_in_) from a literature reference population of untreated fluorochemical manufacturing plant retirees (n = 26, age >55 yrs).

**Results:**

For all three PFAAs monitored during phlebotomy, HL_app_ in each of the family members (except the mother, who had a low rate of venesection) was significantly shorter than the geometric mean HL measured in the reference population, and in some cases were even shorter compared to the fastest eliminator in the reference population.

**Conclusion:**

This study suggests significantly accelerated PFAA clearance with regular phlebotomy treatment, but the small sample size and the lack of controls in this clinical intervention precludes drawing firm conclusions. Given the minimal risks of intermittent phlebotomy, this may be an effective and safe clinical intervention to diminish the body burden of PFAAs in highly exposed people.

## Introduction

Perfluoroalkyl acids (PFAAs) have had many historic commercial and industrial applications, including surface-treatment of furniture, carpets and clothing for stain resistance, packaging for food, mist suppression in metal plating, and in fluoropolymer manufacturing [1], [2]. Due to the wide environmental distribution of PFAAs, human exposure can occur from ingestion of food and water, inhalation of air or dust, vertical transmission to offspring, or dermal absorption from treated commercial products [3].

Perfluorohexane sulfonate (PFHxS), perfluoroctane sulfonate (PFOS) and perfluorooctanoic acid (PFOA) are the most prominent and commonly detected PFAAs in North Americans today, usually with levels in the low parts-per-billion range (i.e. ng/mL in serum) [4], [5], [6], [7]. However, some individuals and distinct subpopulations have much higher PFAA contamination as a result of occupational exposure [8], or localized industrial contamination [9], such as a recent PFOA spill into a drinking water supply [10]. Of direct relevance to the current study, a Canadian family was recently identified with elevated PFAA levels, likely as a result of repeated commercial surface treatment applications to the carpeting within their in-floor heated home [11].

Human elimination half-life (HL), the time required for the amount of a chemical to be reduced by 50% in human serum, is commonly used to describe how fast, or slow, the human body can eliminate a specific compound [12]. This parameter can be difficult to measure for persistent contaminants such as PFAAs [13] because of on-going exposure during the monitored elimination period, as well as fluctuations in body weight (e.g. “growth dilution”). The intrinsic human elimination half-life (HL_in_) is the “true” HL that corrects for these biases, while the apparent half-life (HL_app_) refers to a HL that does not incorporate such confounders. A common way to estimate HL_in_ is to monitor a highly-exposed adult population after the major source of exposure has been removed, because body weight fluctuation is minor in adults and on-going exposure can be ignored because it is insignificant relative to the starting body-burden of the chemical. In highly contaminated retirees (>55 yrs old) that were previously exposed occupationally in a fluorochemical manufacturing plant, Olsen *et al.* [8] estimated HL_in_ to be 8.5 years for PFHxS (95% confidence interval (CI), 6.4–10.6 yrs), 5.4 years for PFOS (95%CI, 3.9–6.9 yrs), and 3.8 years for PFOA (95%CI, 3.1–4.4 yrs).

In a study of urinary excretion in a background population in China, Zhang *et al*. [14] estimated HL_app_ for PFHxS, PFOS, and PFOA to be 7.7, 6.2, and 2.1 years, respectively, for reproductive-aged females, and 35, 27, and 2.6 years, respectively, for males and post-menopausal females. As discussed by Zhang *et al*., significantly faster PFAA elimination in reproductive-aged women is likely due to menstruation [15], gestational transfer [16], [17] and breast feeding [17], [18], [19]. Zhang *et al*. did not consider certain routes of excretion (fecal, sweat, hair, nails etc.), which likely explains the longer PFHxS and PFOS HL_app_ estimates in men and post-menopausal women compared to the HL_in_ estimates from Olsen *et al.* [8].

In animal and human studies, PFAA exposure has been associated with immunotoxicity [20], reproductive toxicity [21], [22], neurotoxicity [23], hepatotoxicity [24], endocrine disruption [25], and some PFAAs are likely human carcinogens [26], [27]. PFAAs also transmit vertically in pregnancy [17] and persist in offspring. Toxicological thresholds of adverse effects in humans are not completely delineated, but possible pathophysiological mechanisms include neurodevelopmental abnormalities [28], mitochondrial damage [29], epigenetic alteration [30], induction of apoptosis [31], impaired hormonal synthesis [32], cell membrane toxicity [33], and tumorigenesis [34]. Some PFAAs such as PFOS and PFOA appear to exert pathophysiological impact at miniscule doses, as low as a few parts per billion. [35] Considering the persistent nature of PFAAs in the human body, and emerging evidence of health risks, increased study of interventions that may hasten the elimination of PFAAs should be considered.

Research to date confirms that some bile acid sequestrants, such as cholestyramine, appear to be successful at facilitating removal of selected PFAAs, including PFHxS, PFOA, and PFOA [36], [37], [38]. Subsequent to widespread population exposure to PFOA following drinking water contamination in West Virginia, Ducatman *et al.* [39] reported that individuals using cholestyramine for other medical reasons generally had significantly lower PFOA levels compared to non-users. Detoxification interventions such as thermal depuration [40], and herbal remedies including zeolites [36], saponins [36], as well as *Chlorella pyrenoidosa* [37] do not seem to be effective at eliminating PFAAs.

Venesection, also known as phlebotomy, is the drawing of blood for the purpose of laboratory investigations, for benevolent blood donation, or for therapeutic indications such as the treatment of hemochromatosis or polycythemia. An Australian study comparing serum PFAA levels of the general population to individuals undergoing intermittent blood withdrawals for medical reasons demonstrated significantly lower PFAA concentrations in those receiving venesection [41]. These results are not entirely surprising because PFAAs have rather low volumes of distribution, meaning they are primarily distributed in the blood and are otherwise most abundant in organs that are heavily perfused by blood, such as liver and kidney [42]. This is partly due to their strong binding to serum proteins [43].

This study is an analysis of PFHxS, PFOS, and PFOA concentrations in serum throughout a 4–5 year prescribed program of intermittent therapeutic phlebotomy in 6 members of a highly-exposed family. The aim was to evaluate if therapeutic phlebotomy helped to accelerate PFAA elimination when performed at intervals similar to, or less frequent than what is done for routine blood donation at Canadian Blood Services. The effectiveness of treatment for each family member was evaluated by comparing the elimination kinetics (HL_app_) from treated family members to the established range of kinetics (HL_in_) from a reference population [8]. Although this was a clinical intervention with no ideal experimental controls to match for age and gender, the results were promising that the low-risk treatment was effective.

## Materials and Methods

### 2.1. Participant information

As part of routine care at an environmental health clinic in Edmonton, Alberta, Canada, patients are commonly tested for toxicant exposures when their detailed life history suggests possible elevated exposure to persistent toxic substances. A panel of testing for assorted compounds at a commercial laboratory, including for PFAAs, is commonly undertaken. Through this process a family was previously identified to have elevated serum concentrations of PFAAs, and a subsequent study of their in-floor heated home suggested that the source of this exposure was repeated commercial spraying of their home carpets with stain-repellants [11]. Specifically, the serum PFHxS level of each family member was in excess of the 95^th^ percentile according to reference data from the US Centers for Disease Control and Prevention, and Health Canada [4], [7]. By the same comparative metrics, PFOS was generally highly elevated, and PFOA was moderately elevated, but the extent of contamination differed among the children and parents [11].

No major health problems were reported by the participants before, during, or after the intervention. One notable concern however was on the part of the mother, who gave an account of 2 consecutive second-trimester pregnancy losses, one with an evident facial deformity in the deceased offspring. Both gestational losses occurred within 3 years of moving into the home that was later discovered to have elevated dust PFAA concentrations. Two prior pregnancies in the previous residence proceeded uneventfully. While no clear link could be made between the PFAA exposures and pregnancy losses, these experiences were nevertheless part of the family members’ motivation to undergo the phlebotomy treatment.

### 2.2. Therapeutic approach

When elevated levels of toxicants are identified in the environmental health clinic, discussion of options to facilitate clearance is undertaken with each patient. As part of the clinical care process in this case, discussion of the recent scientific literature including potential PFAA health risks and their long elimination half-lives was undertaken by the environmental health physician. While no recognized treatment for hastening the elimination of PFAAs in humans was found in the literature at the time this treatment begun, experimental options were discussed to fully inform the patients of potential clinical possibilities.

Consultation was undertaken regarding plasmapheresis, the therapeutic removal of plasma. This procedure, however, was deemed by the participants to be invasive and to potentially introduce new and unwelcome risk factors. For example, as part of the plasmapheresis procedure, intravenous tubing and solution bags with phthalate plasticizers [44] would be introduced to the participants, thus repeatedly exposing them to endocrine-disrupting toxicants [45]. The family members chose not to pursue this potential therapy.

Given the established global safety record associated with benevolent phlebotomy (blood donation), and after the potential risks and benefits of this approach were discussed in detail with each participant in this 7 member family, 6 of the family members (see demographics in Table 1) expressed the desire and intent to undertake regular therapeutic phlebotomy in an attempt to more rapidly diminish their body burden of PFAAs. One family member declined to participate at this point. PFAA concentrations were analyzed immediately after each occasion of venesection to determine the efficacy of the proposed approach. The current study combines all of these data from 4+ years of treatment. Ethics approval for this retrospective study of the combined dataset was received from Health Ethics and Research Board at the University of Alberta.

**Table 1:** Demographic and related information about the study participants.

	*Father*	*Mother*	*1^st^ Child*	*2^nd^ Child*	*3^rd^ Child*	*4^th^ Child*
***Gender***	Male	Female	Male	Female	Male	Male
***Intervention period***	Feb.2,2009–Oct.27,2013	Jun.1,2009–Oct.6,2013	Jun.9,2009–Jul.30,2013	Jun.6,2009–Jul.15,2013	Jun.1,2009–Oct.27,2013	Jun.2,2009–Oct.27,2013
***Time moved out of the house***			Jul.2009	Sept.2010		
***Age when phlebotomy*** ***started (yrs)***	53	47	22	19	17	16
***Body weight during*** ***intervention (kg)***	80	55	65–72	55–58	60–67	55–69
***Percent change in body*** ***weight during intervention***	0%	0%	10.8%	5.4%	11.7%	25.4%

After receiving informed consent, intermittent phlebotomy was undertaken at an overall rate (Table 2) that was equal to or less than the maximum rate of what is done for routine blood donations at the Canadian Blood Services: ∼500 ccs of whole blood every 56 days. Frequency and timing of blood removal was not consistent between family members because of their personal availability, success at each time of venesection, and compliance of each participant. The mother had a much lower rate of blood removal compared to other family members (Table 2) because phlebotomy was often unsuccessful in her as a result of a lack of accessible veins for venesection, unlike the prominent veins found in remaining family members. Nevertheless, the mother’s rate of blood removal by phlebotomy (0.028 mL blood/day/kg) is significant and similar to the average rate of blood loss by menstruation (0.029 mL blood/day/kg) [14], [46], which on its own is suggested to accelerate PFOS clearance in women relative to men [47]. Mineral supplementation was discussed and was taken by each participant.

**Table 2:** Phlebotomy details about the phlebotomy program used in the family.

	*Father*	*Mother*	*1^st^ Child*	*2^nd^ Child*	*3^rd^ Child*	*4^th^ Child*
***Elapsed time between first and last intervention (days)***	1728	1588	1512	1500	1609	1608
***Accumulative whole blood drawn (mL)***	12048	2420	6600	5735	11385	9430
***Average whole blood drawn/day (mL/day)***	7.0	1.5	4.4	3.8	7.1	5.9
***Average whole blood drawn (mL/56 days)*** *	392	84	246	213	398	330

*The maximum blood donation recommended by Canadian Blood Services is ∼500 ml every 56 days.

With the absence of any adverse side-effects, and apparent success at diminishing serum PFAA levels in each case following early blood draws, each participant decided to continue with the intermittent phlebotomy process as their treatment of choice for PFAA clearance. No adverse incidents or negative long term impact were observed or reported by any of the family members as a result of the intervention.

### 2.3. Ongoing PFAA exposure

All carpeting in the home, which was identified as the likely major source of PFAA exposure for the family [11] was removed January 20, 2009. Air purification in the home was also achieved over the next 3 months by the addition of three interventions: i) a medical-grade Hepa filter, ii) carbon filtration to eliminate volatile organic compounds, and iii) improved air exchange ventilation with a heat recovery ventilator. In 2012, analysis of the house dust showed that concentrations had declined at least 5 to 10-fold. Over the years following the commencement of phlebotomy, the first and second child (Table 2) sequentially moved out of the residence at different times to attend schooling or commence work in other locales. No obvious PFAA source was identified in their new residences or workplaces.

### 2.4. PFAA analysis

**Figure 1:**
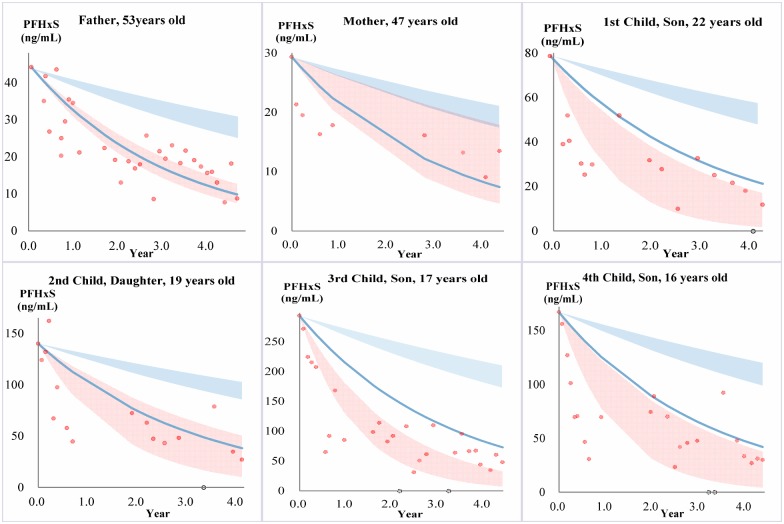
Serum PFHxS elimination in each family member. PFHxS elimination in family members undergoing phlebotomy (red) compared to a reference population of occupationally exposed retirees (blue). Red data points are measured PFHxS concentrations in the family members, and the red band is the calculated 95% CI of a fitted exponential decay model. The blue band represents the 95% confidence interval of the geometric mean PFHxS HL_in_ (5.8–9.2 yrs) for the reference population, while the blue line represents the fastest eliminator in the same reference population (HL_in_ = 2.2 yrs). Black hollow circles indicate a phlebotomy session where the serum sample was not analyzed, and therefore was not used in calcualtions.

**Figure 2:**
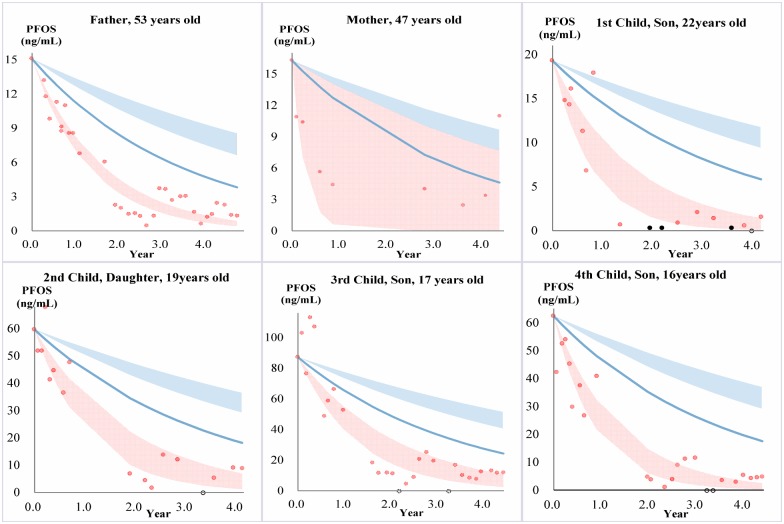
Serum PFOS elimination in each family member. PFOS elimination in family members undergoing phlebotomy (red) compared to a reference population of occupationally exposed retirees (blue). Red data points are measured PFOS concentrations in the family members, and the red band is the calculated 95% CI of a fitted exponential decay model. The blue band represents the 95% confidence interval of the geometric mean PFOS HL_in_ (4.0–5.8 yrs) for the reference population, while the blue line represents the fastest eliminator of PFOS in the same reference population (HL_in_ = 2.4 yrs). Black hollow circles indicate a phlebotomy session where the serum sample was not analyzed, and therefore was not used in calcualtions. The black dots represent measurements which were below the LOQ 0.5 ng/mL; and LOQ/√2 (0.35 ng/mL) was used for the purpose of statistical analysis and plotting.

**Figure 3:**
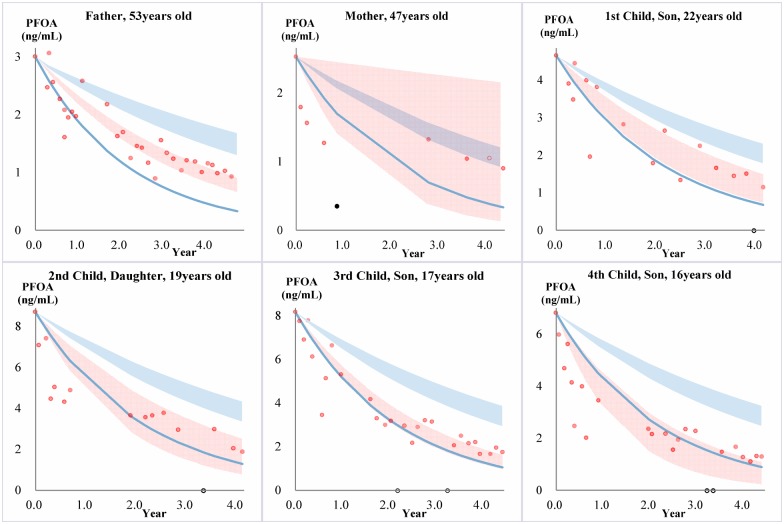
Serum PFOA elimination in each family member. PFOA elimination in family members undergoing phlebotomy (red) compared to a reference population of occupationally exposed retirees (blue). Red data points are measured PFOA concentrations in the family members, and the red band is the calculated 95% CI of a fitted exponential decay model. The blue band represents the 95% confidence interval of the geometric mean PFOA HL_in_ (3.0–4.1 yrs) for the reference population, while the blue line represents the fastest eliminator of PFOA in the same reference population (HL_in_ = 1.5 yrs). Black hollow circles indicate a phlebotomy session where the serum sample was not analyzed, and therefore was not used in calcualtions.

All serum samples were submitted to ALS Laboratories (Edmonton, AB) for quantitative analysis of PFAAs (see Supporting Information, S1 Table). Samples were extracted by solid phase extraction and concentrations of PFHxS, PFOS, and PFOA were determined using high performance liquid chromatography/tandem mass spectrometry. Formic acid (0.1 M) along with a mixture of isotopically labeled PFAAs (Wellington Laboratories, Guelph, Ontario) were added to 1.0 mL of serum. The mixture was vortexed, sonicated, and subjected to solid phase extraction. The extract was concentrated to 100 uL and an instrument performance internal standard was added along with 200 uL of 90% 20 mM aqueous acetic acid/10% methanol. Procedural blanks were run with each set of samples, and method limits of quantification (LOQ) were 0.5 ng/mL for each analyte. For the few PFAA levels that fell below this LOQ (shown as hollow circles in Figs. 1–3), LOQ/√2 (i.e. 0.35 ng/mL) was substituted for purposes of data analysis. PFHxS, PFOS, and PFOA levels in all participating family members were >LOQ throughout the intervention except for 3 PFOS measurements out of 16 total measurements in the 3^rd^ child and one PFOA measurement out of 9 total measurements in the mother. Written informed consent was given by participants (or parents in the case of children) for their clinical records to be used in this study.

### 2.5. PFAA kinetic modeling and HL_app_ calculation

With about 4 years of follow-up, decreasing PFAA trends were observed in each family member. To evaluate the effect of phlebotomy, versus the expected rate of intrinsic elimination, we graphically and statistically compared the observed decreasing trends in each family member (fitted to a simple exponential decay model) with reference population [48] elimination kinetics. Specifically, for the reference population of occupationally exposed retirees, we considered: i) the 95% confidence interval of the geometric mean elimination rate for each PFAA, and ii) the fastest individual rate of elimination rate among the reference population. For comparative purposes, the concentration of each PFAA in serum on the first day of the intervention was taken as time zero, and the models were forced through this starting point.

One metric that was calculated to compare the efficiency of the phlebotomy for removal of different PFAAs in each family member was termed the phlebotomy efficiency coefficient (EC). This was based on the subtractive difference between HL_app_ (yrs) in each treated family member and the geometric mean HL_in_ (yrs) of the reference population according to **Equation 1**.
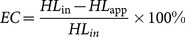
(1)


## Results and Discussion

### 3.1 PFAA elimination trends and HL_app_ in the family

When comparing PFHxS data for the participants with the 95^th^ centile among Canadians in 2009–2011 (8.9 ng/mL) [7], all family members had much higher starting concentrations (Table 3). For example, the 4^th^ son had PFHxS almost 20-fold higher. For PFOS, the 3 youngest children had starting serum concentrations 3–5 fold higher than the 95^th^ percentile (18 ng/mL) among Canadians, while the parents and the 1^st^ child had starting PFOS levels comparable to this 95^th^ percentile. For PFOA, all children were comparable to the 95th percentile (5 ng/mL) at the beginning of the intervention, whereas the parents were comparable to the median (2.7 ng/mL) among Canadians.

**Table 3:** PFAAs serum concentrations before and after the treatment by phlebotomy for each participant.

		*Father*	*Mother*	*1^st^ Child*	*2^nd^ Child*	*3^rd^ Child*	*4^th^ Child*	*Average*
***PFHxS***	Before	44.1	29.3	78.6	140	293	167	125
***(ng/mL)***	After	8.8	13.5	11.9	27.1	48.3	29.9	23.3
***PFOS***	Before	15.1	16.3	19.3	59.7	87.3	62.4	43.4
***(ng/mL)***	After	1.4	11.0	1.6	9.0	12.2	5.0	6.7
***PFOA***	Before	3.0	2.5	4.7	8.7	8.2	6.8	5.7
***(ng/mL)***	After	1.2	0.9	1.1	1.9	1.8	1.3	1.4

For comparison, the 95th percentiles for PFHxS, PFOS and PFOA were 8.9, 18.0 and 5.0 ng/mL, respectively, among Canadians for the period 2009–2011 [7].

There were clear declines in PFAA levels in the family members over the ∼4 years of treatment with intermittent phlebotomy (Table 3). By the end of testing in the fall of 2013, average PFHxS had declined from 125 ng/mL to 23.3 ng/mL, average PFOS from 43.4 ng/mL to 6.7 ng/mL, and average PFOA from 5.7 ng/mL to 1.4 ng/ml.

**Figure 4:**
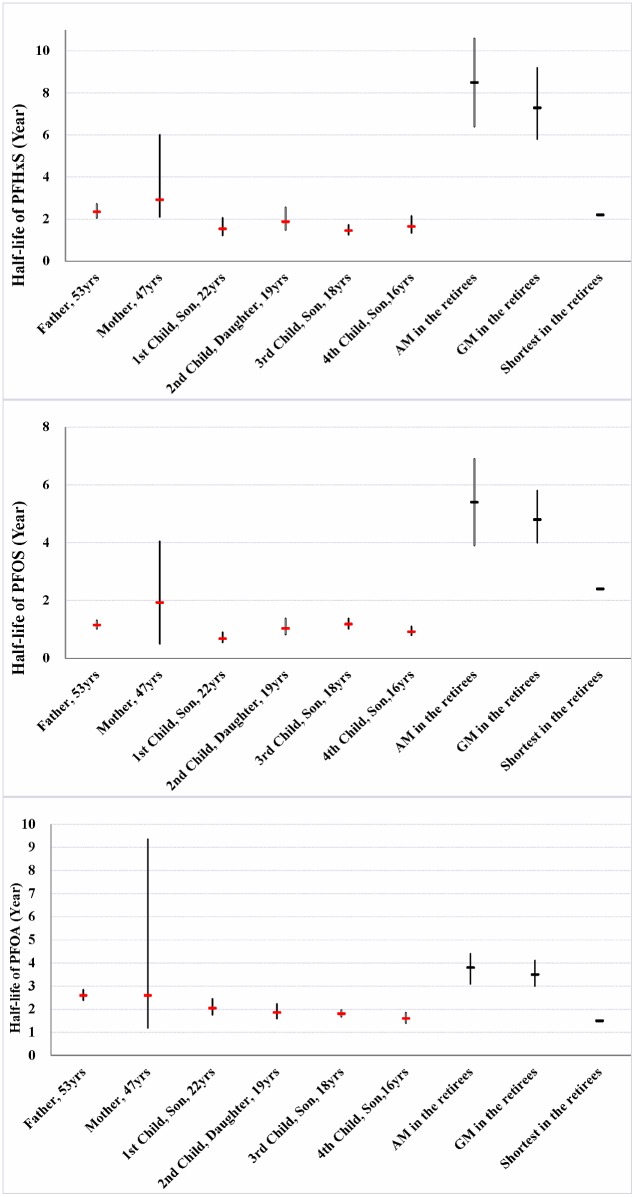
Apparent elimination half-lives (HL_app_) of PFHxS, PFOS and PFOA in the family. Calculated HL_app_ in the family members based on all measurements in the current study (red markers). Black markers are HL_in_ from the reference population of Olsen *et al*.: arithmetic mean, geometric mean, and shortest among all individuals. Black error bars are the associated 95% confidence intervals.

For PFHxS, comparing to the reference population geometric mean elimination kinetics (Fig. 1, blue shaded regions show the 95^th^ confidence interval for reference population), the fitted elimination for most family members (Fig. 1, red shaded region shows 95^th^ confidence interval for each participant in phlebotomy), except for the mother, was significantly faster (i.e. no overlap of the blue and red shaded regions) [8]. Correspondingly, the measured HL_app_ in most family members, except for the mother, was significantly shorter than the geometric mean HL_in_ for the reference population (Fig. 4). The mother did not have a significantly faster PFHxS elimination rate, compared to the reference population, likely owing to difficulties with performance of venesection that resulted in a much lower rate of blood removal (Table 2).

Taken together, these results are suggestive that phlebotomy was effective for PFHxS removal, and that the rate of phlebotomy was a determining factor (i.e. the mother’s rate of elimination was slower, as was her rate of phlebotomy). Nevertheless, the absence of experimental control, e.g. where serum concentrations might have been monitored in each participant for a year before introducing phlebotomy (which was not deemed ethically advisable here), makes it difficult to make firm conclusions. Among the reference population (26 individuals) there was one individual outlier that eliminated PFHxS comparably fast (HL_in_ = 2.2 yrs), which was in the range of the HL_app_ for all participants receiving phlebotomy here (Fig. 4, and blue lines in Fig. 1). It is also important to consider that the reference population in Olsen’s study [8] were all >55 years old and comprise 92% males, and thus are also not a perfect reference population, but are the best data available in the literature.

For PFOS the results were very similar to PFHxS, showing significantly faster rates of elimination compared to the reference population at the geometric mean level (except for the mother due to fewer blood draws), and elimination rates were even faster compared to the fastest PFOS eliminator in the reference population (Fig. 2 and Fig. 4).

For PFOA, the observed declines were also faster than HL_in_ for the reference population, except for the mother (Fig. 3), but differences were generally not as significant as for either PFHxS or PFOS (Figs. 1–3 and Fig. 4). In fact, compared to the fastest eliminator in the reference population, the observed trends were comparable or even slower (e.g. data for the father). At the geometric mean level, only the children had faster elimination compared to the reference population (Fig. 3 and Fig. 4).

### 3.2 Phlebotomy efficiency

**Figure 5:**
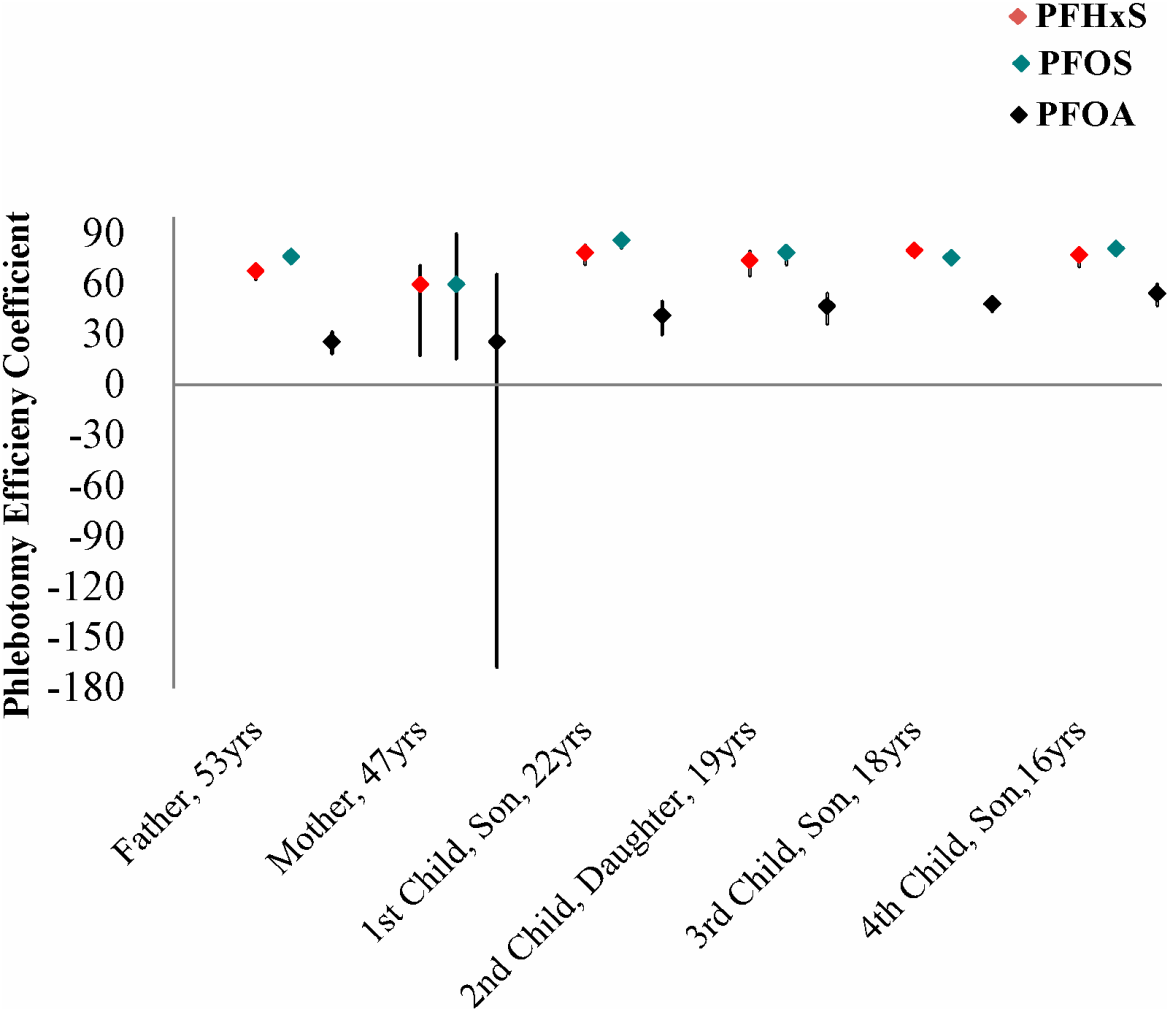
Phlebotomy efficiency coefficients for PFHxS, PFOS and PFOA in the family. Black error bars represent the 95%CI of each calculated half-life.

From phlebotomy efficiency coefficients (Fig. 5), it was evident that the effect of phlebotomy was most pronounced for PFOS and PFHxS, relative to PFOA. PFOS and PFHxS are naturally cleared much more slowly than PFOA, [8] thus this trend with phlebotomy is expected and can be taken as further evidence that phlebotomy was an effective mode of elimination for the most persistent PFAAs. Nevertheless, there are limitations in our interpretation. First, it is difficult to know for the current participants how much bias there was in PFOA HL_app_ estimates due to background exposure (which would bias the estimates towards longer HL_app_) since the starting PFOA concentrations in the family were not very high (i.e. not above the 95^th^ percentile among Canadians). [7] In addition, when considering possible age or gender differences, and the comparison to a mostly older and male reference population, there may be some uncertainty in the validity of comparison.

### 3.3. Synopsis of Study Results

- All family members (except for the mother) with regular phlebotomy intervention had significantly shorter HL_app_ for PFHxS, PFOS and PFOA than the geometric mean HL_in_ in the reference population [8].
- Some family members undergoing phlebotomy treatment had even shorter HL_app_ for PFHxS, PFOS and PFOA than the fastest HL_in_ measured among all untreated fluorochemical-plant retirees [8].
- The phlebotomy efficiency for enhancing PFAA elimination followed the order of PFOS≈PFHxS>PFOA, consistent with persistence of the three PFAAs in the human body, [8] suggesting that the more biopersistent PFAAs are most effectively eliminated by phlebotomy.
- Using the mother as a comparison, whom received the lowest rate of phlebotomy, the rate of decrease in serum PFAA levels generally paralleled the total volume of blood drawn, suggesting a dose-response effect for the treatment.
- There was some variation in elimination rates between participants that did not parallel the rate of blood removal. The HL_app_ during phlebotomy, for example, appeared to be more rapid among the children compared with the father, which may be attributed to a faster HL_in_ for younger individuals, and perhaps a minor growth-dilution effect.

### 3.4. Suitability of this clinical approach

Some may question whether it is apposite to use clinical interventions to eliminate toxicants such as PFAAs when there are no established clinical diagnoses, to date, that are confirmed to be the direct consequence of PFAA exposure. When considering intervention in any clinical case, the totality of risks, benefits, patient objectives, informed consent, compliance, standard-of-care, knowledge translation, as well as quality and integrity of research evidence, [49], [50] must all be incorporated into optimal clinical decision-making. [51], [52] Furthermore, it is well established that there is enormous lethargy in translation of emerging scientific evidence into clinical practice [51], [53]. After reviewing the body of contemporary toxicology literature, the participants in this case series in conjunction with their health provider made the informed clinical decision to proceed with intervention based on various factors.

As discussed in the introduction, there is considerable literature suggesting pathophysiological associations with exposure to PFAAs in animal models, and in humans. Full consensus of the adverse impacts of toxicants, and of the respective safety thresholds, typically takes decades of research and observation. New evidence for potentially irreversible effects of PFAAs is now being reported [3], [27].

It is difficult to conclusively attribute specific diagnoses and illnesses to a single toxicant because of multiple confounders in clinical situations. Direct links to specific diagnoses are therefore challenging to establish. This challenge, however, does not minimize the reality of serious potential sequelae associated with PFAAs exposure. With informed awareness of uncertainty and potential toxicant effects, contaminated patients have the right to make informed decisions about the approach to their clinical care. Respecting patient autonomy is a hallmark of medical ethics. Moreover, the chosen intervention of intermittent phlebotomy has an established record of minimal risk and enormous safety. Potential benefits may outweigh risks by a considerable margin. Accordingly, rather than waiting for affliction to befall individuals and populations, some health practitioners consider it unethical to **not** offer safe interventions whenever possible in clinical situations to preclude adverse health effects in the face of recognized toxicant exposures.

Phlebotomy is not being advocated in this paper as a detoxification intervention for all toxicant exposures. Most bioaccumulative toxicants, for example the brominated flame retardants [54], organic solvents [55], chlorinated pesticides [56], and polychlorinated biphenyls [57], are lipophilic and accumulate to a great extent in body compartments other than blood. Clearance of persistent lipophilic compounds may require specific interventions which mobilize toxicants from their tissue storage sites and subsequent interruption of the enterohepatic circulation [58], [59] to prevent reabsorption.

### 3.5. Limitation of this clinical study

There are limitations in this study which must again be acknowledged. First, the retrospective case study has a small sample size and all participants are from one family, making generalizations to other populations difficult where wider genetic variability will be present. Future studies with a larger sample size from different population groups would provide more reliable evidence in terms of phlebotomy efficiency and safety. Second, the absence of perfect experimental controls is a limiting feature. Future studies with controls (e.g. self-controls, to compare PFAA levels within the same individual before and after the intervention phlebotomy) would be of great value in establishing phlebotomy efficiency.

## Conclusion

According to the results of this study, intermittent phlebotomy in line with a typical schedule used by blood donation services appears to be a safe and effective means to facilitate removal of bioaccumulated PFHxS, PFOS and possibly PFOA (although data are weakest for the latter). In view of the potential benefits and minimal risks of this intervention, it is suggested that intermittent phlebotomy at the same rate and schedule that is used for blood donation, be considered as a clinical intervention for individuals found to have marked elevation of any or all of PFHxS, PFOS and PFOA. Incorporating other PFAA clearance research, phlebotomy as an intervention might be used alone or in conjunction with bile acid sequestrants [36], [40] in order to hasten PFAA elimination in clinical situations. At this point, however, it has not yet been determined which serum threshold level for any of the PFAAs compounds poses a significant risk for adverse health sequelae in relation to fetal development, in the pediatric population, or in adults. Accordingly, clinical judgment in conjunction with informed patient consent should be used when considering interventions to facilitate removal of PFAAs. Removal of PFAAs may be particularly important to prevent vertical transmission in reproductive-aged woman in order to minimize future risks of adverse pediatric health outcomes as a result of intrauterine exposure to the fetus.

## Supporting Information

S1 Table **PFAA data from participants undergoing phlebotomy treatment.**
(XLSX)
